# Monocyte-Derived Dendritic Cells Exhibit Increased Levels of Lysosomal Proteolysis as Compared to Other Human Dendritic Cell Populations

**DOI:** 10.1371/journal.pone.0011949

**Published:** 2010-08-02

**Authors:** Nathanael McCurley, Ira Mellman

**Affiliations:** 1 Departments of Cell Biology and Immunobiology, Ludwig Institute for Cancer Research, Yale University School of Medicine, New Haven, Connecticut, United States of America; 2 Emory Vaccine Center and Department of Pathology and Laboratory Medicine, Emory University, Atlanta, Georgia, United States of America; 3 Genentech, South San Francisco, California, United States of America; New York University, United States of America

## Abstract

**Background:**

Fine control of lysosomal degradation for limited processing of internalized antigens is a hallmark of professional antigen presenting cells. Previous work in mice has shown that dendritic cells (DCs) contain lysosomes with remarkably low protease content. Combined with the ability to modulate lysosomal pH during phagocytosis and maturation, murine DCs enhance their production of class II MHC-peptide complexes for presentation to T cells.

**Methodology/Principal Findings:**

In this study we extend these findings to human DCs and distinguish between different subsets of DCs based on their ability to preserve internalized antigen. Whereas DCs derived *in vitro* from CD34+ hematopoietic progenitor cells or isolated from peripheral blood of healthy donors are protease poor, DCs derived *in vitro* from monocytes (MDDCs) are more similar to macrophages (MΦs) in protease content. Unlike other DCs, MDDCs also fail to reduce their intralysosomal pH in response to maturation stimuli. Indeed, functional characterization of lysosomal proteolysis indicates that MDDCs are comparable to MΦs in the rapid degradation of antigen while other human DC subtypes are attenuated in this capacity.

**Conclusions/Significance:**

Human DCs are comparable to murine DCs in exhibiting a markedly reduced level of lysosomal proteolysis. However, as an important exception to this, human MDDCs stand apart from all other DCs by a heightened capacity for proteolysis that resembles that of MΦs. Thus, caution should be exercised when using human MDDCs as a model for DC function and cell biology.

## Introduction

The role of macrophages (MΦs) in the acquisition and degradation of exogenous material is well established throughout the phylogeny of metazoans [1]. Yet in vertebrates such complete degradation is inconsistent with the production of peptides of sufficient length (13–17 amino acids) to bind class II MHC molecules for presentation to T cells [2], [3]. Antigen processing requires *limited* degradation of proteins and preservation of cognate T cell epitopes [4]. It was recently demonstrated in mice that the most efficient antigen presenting cells, dendritic cells (DCs) and B cells, are distinguished from MΦ in their ability to greatly attenuate lysosomal degradation of internalized antigen [5], [6]. This is mechanistically mediated through a fine control of lysosomal proteolytic activity that was previously unappreciated. Both DCs and B cells, *in vitro* and *in vivo*, exhibit a remarkably low level of lysosomal protease expression. DCs furthermore control degradation by modulation of lysosomal pH that attenuates proteolysis in the immature state and moderately increases the level of proteolysis with maturation [7]. Additionally, in the case of phagocytosed antigens it has been demonstrated that NOX2 contributes to an increase in the alkalinity of the phagolysosome, further limiting proteolysis [8], [9].

Both mouse and human DCs found *in vivo* have been categorized into a number of subsets based on phenotypic and functional differences [10], [11]. Moreover, several methods have been developed for deriving subsets of human DCs *in vitro* from precursor cells, most commonly from CD34^+^ hematopoietic precursors (CD34DCs) and monocytes (MDDCs). CD34DCs have the advantage of being derived from an early hematopoietic precursor (analogous to bone marrow-derived DCs [BMDCs] in mice), though the number of starting cells can be limiting. On the other hand, monocytes are an abundant cell type from which large numbers of MDDCs can be cultured, though they are more derived precursors which are already committed to the monocyte/MΦ linage. In the study that follows we extend the initial investigations of lysosomal function in mouse DCs to both *in vivo-* and *in vitro*-derived DCs of human origin.

## Results

### MDDCs are distinguished from other DC subsets in having high lysosomal protease content

**Figure 1:**
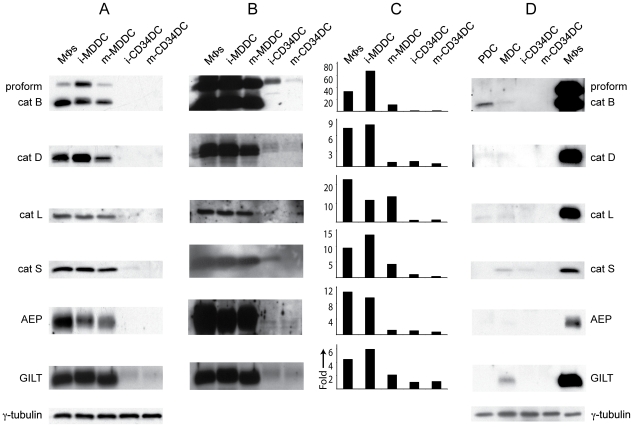
MDDCs are abundant in lysosomal proteases compared to other DCs. (A) Immature (i-) and mature (m-) MDDCs are comparable to MΦs in protease protein abundance as assessed by immunoblot of cell lysates. By contrast, immature and mature CD34DCs exhibit remarkably lower expression levels of protease protein than either MΦs or MDDCs. (B) Overexposure of the blots from (A) reveals that the enzymes are present in CD34DCs, though in strikingly diminished amounts. (C) Quantitative RT-PCR shows that MΦs and MDDCs are also distinct in having a high quantity of transcripts for the enzymes compared to CD34DCs. Data are displayed as “fold-greater” than immature CD34DCs. (D) PDCs and MDCs taken *ex vivo* from healthy donors also display markedly low levels of lysosomal protease expression. γ-tubulin was used as loading control.

We first investigated the relative abundance of representative lysosomal proteases in human monocyte-derived MΦs, MDDCs, and CD34DCs. These cells were cultured as previously described [12], [13] and cell-free extracts were prepared for immunoblot analysis of the proteases and γ-interferon-inducible lysosomal thiol reductase (GILT). Surprisingly we found that cathepsins (cat) B, D, L and S, asparginyl endopeptidase (AEP), and GILT were in near equal abundance in MΦs and immature MDDCs, slightly less abundant in populations of mature MDDCs (produced by overnight treatment with LPS), with only trace amounts present in CD34DCs (Fig. 1A). Overexposure of the blots revealed that these enzymes were present in CD34DCs, though in markedly lower abundance (Fig. 1B).

To assess whether the differences in lysosomal protease expression could be accounted for at the transcriptional level, we performed quantitative RT-PCR on RNA samples from MΦs, MDDCs, and CD34DCs using primers for catB, catD, catL, catS, AEP, and GILT. The transcriptional profiles mostly segregated into two distinct groups: the MΦs and immature MDDCs with a high relative level of protease transcription and the immature and mature CD34DCs with a low level of transcription (Fig. 1C). Indeed, a general correlation between the abundance of protease transcripts and protein for these two groups was evident. The transcriptional profile for the mature MDDCs, however, was not proportional to the protein profile, as the level of transcription was closer to that of the CD34DCs, while the amount of protein present more closely matches the MΦs and immature MDDCs. The relative abundance of protease expression at the protein level in mature MDDCs likely reflects the fact that transcription of many genes is reduced following DC maturation but that lysosomal proteases are relatively long-lived.

Given the dramatic differences in protease expression between DCs derived *in vitro* from monocytes and from CD34^+^ hematopoietic progenitor cells, we assessed the protease expression profile of dendritic cells taken *ex vivo* from human blood. Cell-free extracts were prepared from myeloid DCs (MDCs) and plasmacytoid DCs (PDCs) that were purified from the blood of healthy donors as previously described [14]. Both MDCs and PDCs exhibited levels of protease expression that were very low, comparable to CD34DCs, and in marked contrast to MΦs (Fig. 1D).

Taken together these data confirm that DCs most commonly, but not always, contain a low level of lysosomal proteases. While human CD34DCs, PDCs and MDCs share the protease expression characteristics of murine BMDCs and DCs from mouse secondary lymphoid organs, human DCs derived from monocytes are distinguished by a protease expression profile similar to that of MΦs with whom they share a direct precursor (*i.e.*, the monocyte).

### MDDCs exhibit high levels of lysosomal proteolysis *in vitro* compared to CD34DCs

**Figure 2:**
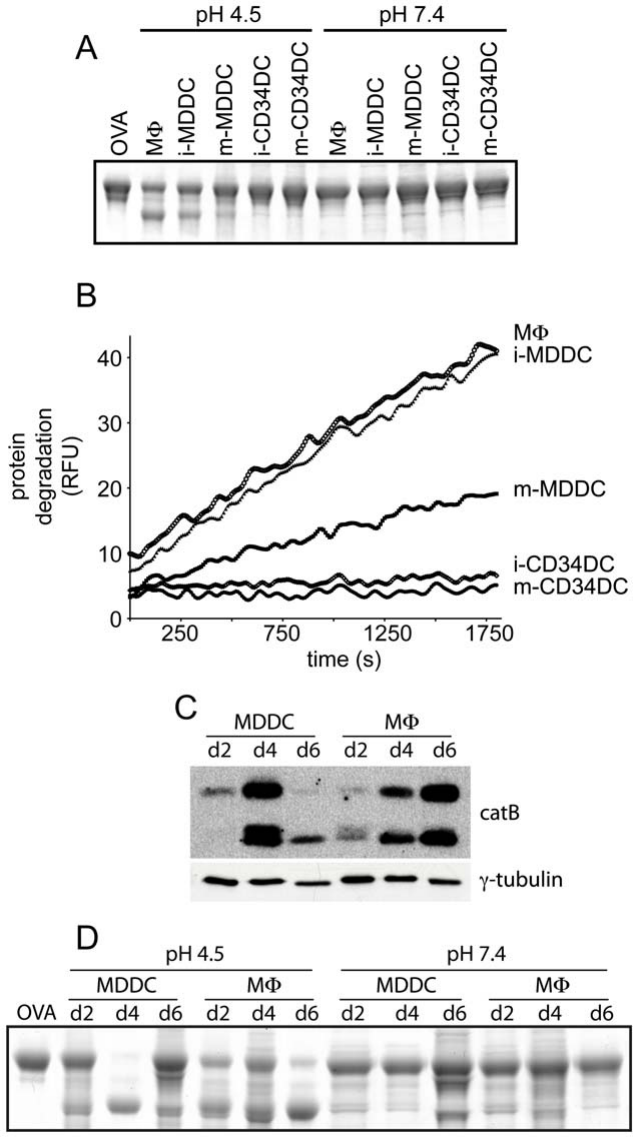
MDDCs exhibit high lysosomal protease activity *in vitro* compared to CD34DCs. (A) Cell lysates prepared from cultures of MΦs, MDDCs, and CD34DCs were incubated together with OVA in either degradation reaction buffer (pH 4.5) or control buffer (pH 7.4). A sample containing OVA in reaction buffer with no lysate was loaded in the first lane as a non-degraded sample. Partial degradation of OVA by MΦs and MDDCs is evident while no degradation by CD34DCs is seen. (B) Quantitation of the rate of degradation by these cells using a self-quenching fluorescent protein substrate demonstrates that immature (i-) MDDCs are equivalent to MΦs in proteolytic capacity, mature (m-) MDDCs are 2-fold less proteolytic than MΦs, while i-CD34DCs and m-CD34DCs are 17- and 28-fold less proteolytic than MΦs, respectively. (C) Cell lysates were prepared from monocyte cultures as they differentiated into either MDDCs or MΦs and were analyzed by immunoblot for catB. CatB expression in MDDCs culminates at day 4 and is diminished following maturation on day 5 and analysis on day 6. MΦs exhibit a steady increase in catB expression from a low level at day 2 to a high level at day 6. (D) Cell lysates of culture samples from (C) were assessed for degradative capacity by incubation with OVA in either reaction buffer (pH 4.5) or control buffer (pH 7.4). Degradation at pH 4.5 correlates with protease expression levels. Relative fluorescence units (RFU).

We next determined whether the observed differences in protease expression were reflected in the proteolytic capacity of MDDCs and CD34DCs. Initial results from *in vitro* degradation assays of OVA protein suggested that MDDCs hydrolyzed proteins at a level matching that of MΦs, while CD34DCs were attenuated in this capacity (Fig. 2A). Lysosomal proteolysis by these cells was quantitatively assessed using an *in vitro* kinetic degradation assay which demonstrated that immature MDDCs degraded the protein substrate at a rate equivalent to that of MΦs, while immature and mature CD34DCs exhibited a 17-fold and 28-fold lower level of degradation than MΦs, respectively (Fig. 2B). The mature MDDCs displayed an intermediate rate of degradation that was 2-fold less than MΦs. Thus the high level of protease expression in MDDCs was reflected *in vitro* by greater degradative capacity.

As described below, developmental upregulation of protease expression was evident in both MDDCs and MΦs derived *in vitro* from monocytes. Cell-free extracts were prepared from monocyte cultures at defined intervals as the cells differentiated into either MDDCs or MΦs. Using catB as a surrogate for the proteases, immunoblotting of these samples revealed that at an early time point of differentiation (day 2) the level of lysosomal protease expression was fairly low (Fig. 2C). CatB expression cumulatively increased in the MΦs on days 4 and 6. In MDDC cultures the level of protease expression on day 4 was roughly equivalent to day 6 MΦs. Maturation of the MDDCs and analysis of the cell extracts on day 6 demonstrated a decrease in protease expression. Again, the level of protease expression correlated with degradative capacity as measured by OVA degradation *in vitro* (Fig. 2D).

### MDDCs and CD34DCs are comparable in lysosomal degradation of non-protein substrates

**Figure 3:**
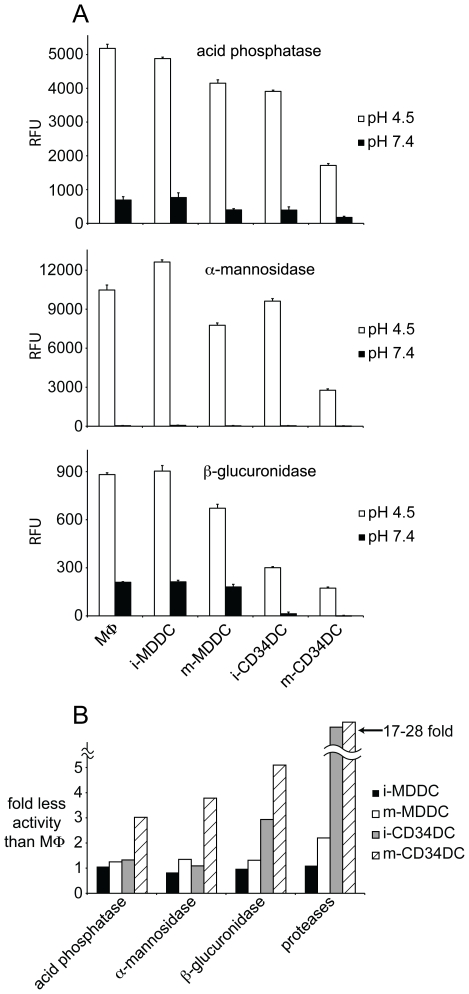
MDDCs and CD34DCs are similar in activity of other lysosomal hydrolases. (A) Cell lysates made from cultures of MΦs, MDDCs, and CD34DCs were incubated together with fluorescent substrates specific for acid phosphatase, α-mannosidase, and β-glucuronidase in either reaction buffer (pH 4.5) or control buffer (pH 7.4). After a 60-minute reaction, detection of the reaction product was measured with a fluorescence spectrophotometer. (B) Compendium of lysosomal hydrolase activity measurements relative to MΦs. While the non-protease acid hydrolases show a similar magnitude of activity in MDDCs and CD34DCs, the differences in proteolysis between the two subsets are accentuated. Immature (i-) and mature (m-) DCs; relative fluorescence units (RFU).

The initial investigation of lysosomal degradation in DCs of mice demonstrated that, in contrast to proteolysis, DCs were comparable to MΦs in lysosomal degradation of non-protein substrates [5]. Indeed, this finding is consistent with the observation that post-translational modifications of proteins only rarely contribute to the cognate T cell epitopes bound to class II MHC [15], [16], [17], perhaps because these modifications are removed in lysosomes. We therefore investigated whether the attenuated proteolytic capacity of human CD34DCs was due to an overall decrease in lysosomal hydrolytic activity or whether it was protease-specific. Cell-free extracts were prepared from MΦs, MDDCs, and CD34DCs and were tested against substrates specific for the activity of lysosomal acid phosphatase, β-glucuronidase, and α-mannosidase. In contrast to the marked difference in protease activity between MDDCs and CD34DCs, these other lysosomal hydrolases were comparable in activity between the two DC subsets (Fig. 3A). Though the greatest difference in hydrolytic activity was seen between the mature CD34DCs and the immature MDDCs when assaying for β-glucuronidase activity, this difference was at most 5-fold, substantially less then the 28-fold difference in protease activity between these two DC types (Fig. 3B). The difference in lysosomal hydrolytic capacity between MDDCs and CD34DCs was therefore predominantly limited to proteolysis, analogous to our previous findings using bone marrow-derived mouse DCs vs. mouse macrophages [5].

### Exogenous antigen is rapidly degraded by MDDCs and preserved by CD34DCs

**Figure 4:**
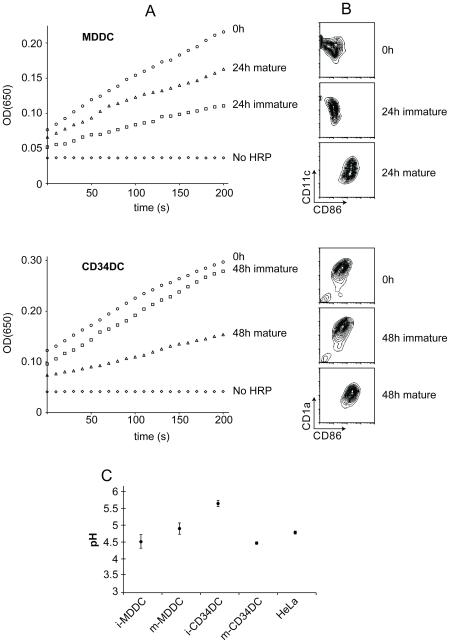
Degradation of internalized proteins by intact MDDCs and CD34DCs. (A) MDDCs (top) or CD34DCs (bottom) were pulse-chased with HRP immune complexes. Immediately after chase (0 h) an aliquot of the cells was measured for HRP activity. The remaining cells were placed back into culture for 24 h (MDDCs) or 48 h (CD34DCs) either in the presence or absence of a maturation stimulus; culture times corresponded to the minimum required to achieve complete (>90%) maturation for each cell type. After this re-culture, the cells were measured for HRP activity. DCs not pulsed with HRP immune complexes (No HRP) were used as negative control and make clear that endogenous peroxidase activity is negligible compared to HRP activity. HRP activity in immature MDDCs is reduced by >50% after 24 h in culture, whereas maturation of the MDDCs attenuates the degradation of HRP. In contrast, even after 48 h in culture, HRP activity in immature CD34DCs remains nearly equivalent to the freshly loaded cells. Maturation of the CD34DCs leads to 50% reduction in HRP activity after 48 h. (B) Maturation of MDDCs (top) or CD34DCs (bottom) that were loaded with HRP immune complexes was assessed by CD86 expression. Immature DCs analyzed either immediately after pulse-chase with HRP immune complexes or following re-culture of the pulse-chased cells were CD86^lo^. DCs that were matured in culture were CD86^hi^. (C) Lysosomal pH of MDDCs and CD34DCs was measured using the pH-sensitive lysosomotrophic dye LysoSensor Yellow/Blue DND-160. HeLa cells are included as reference. Whereas lysosomal pH of MDDCs is low in the immature state and rises slightly with maturation, immature CD34DCs have an elevated lysosomal pH which drops considerably with maturation to a level more conducive to proteolysis. Data are displayed as mean ± s.e.m. Immature (i-) and mature (m-) DCs.

To determine whether antigen was degraded in intact MDDCs and CD34DCs as well as in cell-free preparations, we developed an assay for assessing protein degradation in live DCs. MDDCs and CD34DCs were pulsed for 2 hrs with immune complexes of HRP and polyclonal anti-HRP antibodies, washed, and then returned to culture for 24–48 hr in the presence or absence of a maturation stimulus. After these incubations, the cell-associated HRP activity was determined using a kinetic assay. Intriguingly, while the immature MDDCs displayed an expected loss of HRP activity due to lysosomal degradation, the MDDCs that were matured showed only a modest level of HRP degradation (Fig. 4A and 4B). This was likely due to the decrease in lysosomal protease content as well as an increase in lysosomal pH (see below) that occurred during MDDC maturation. Conversely, even 48 hours after loading the immature CD34DCs, the internalized HRP displayed a minimal amount of degradation. Maturation of the CD34DCs resulted in an increase in lysosomal degradation, presumably reflecting a decrease in lysosomal pH that accompanies maturation (see below).

In an independent set of experiments we measured lysosomal pH of MDDCs and CD34DCs. These studies revealed that lysosomal acidification in CD34DCs was regulated in response to maturation stimuli, as found previously for mouse bone marrow-derived DCs (Fig. 4C). In the immature state, the lysosomes of human CD34DCs exhibited an elevated pH (∼5.6). Given the strict acid requirement for lysosomal proteolytic activity, such an elevated lysosomal pH would result in a significant reduction in the activity of the proteases present. Following LPS-induced maturation of these cells, lysosomal pH dropped closer to the pH optimum of most lysosomal hydrolases (∼4.5) therefore providing an environment more conducive to proteolysis. MDDCs exhibited a low lysosomal pH in the immature state (∼4.5), similar to that found in macrophages (pH 4.7–4.8 [18]) and most other cells.

Two general conclusions can be inferred from this set of data. First, as in the case of murine DCs, human DCs also exhibit a markedly reduced capacity for antigen proteolysis. Second, there is an important exception to this conclusion. MDDCs, the widely used model for DCs derived directly from monocytes, are indeed far more reminiscent of macrophages with respect to their capacity for lysosomal proteolysis than they are similar to DCs, either human or mouse, conventional or plasmacytoid.

## Discussion

DCs were originally identified by their remarkable capacity to stimulate antigen-specific T cell proliferation [19], [20], [21]. Investigation into the mechanisms underlying this capacity revealed that these cells utilize a number of cell biological specializations to achieve this end [4], [22]. In addition to the phenotypic changes that occur with DC maturation and the tight regulation of MHC expression and distribution, recent work has shown that these cells are acutely distinguished from other myeloid leukocytes by specializations in antigen handling and processing within lysosomes [23], [24].

Consistent with the discovery of restricted lysosomal proteolysis in DCs of mice, lysosomes of human DCs taken from blood or derived from hematopoietic progenitors harbor a protease poor, antigen-preserving environment. The combination of low protease content and attenuated lysosomal pH in immature CD34DCs leads to limited degradation of internalized antigen. Concomitant with maturation, lysosomal pH drops and degradation increases. Thus, in addition to well-established mechanisms for antigen acquisition and T cell stimulation, human DCs also utilize mechanisms for antigen preservation that are similar to those of mice.

DCs derived *in vitro* from human monocytes are set apart from other DCs by resembling MΦs in lysosomal degradative capacity. The details of DC ontogeny are under active investigation and the current data indicate that in steady state conditions dendritic cells and monocytes arise from a common precursor cell, while under inflammatory conditions dendritic cells differentiate directly from newly immigrated monocytes [25], [26], [27]. Our data suggest that monocytes have already engaged a developmental program that gives rise to cells with high protease content and that as monocytes differentiate into DCs they acquire many of the characteristic phenotypic traits of DCs while also developing MΦ-like lysosomes. One can distinguish between different subsets of DCs based on functional and phenotypic variation [25] and the presence of highly degradative lysosomes in MDDCs points to a cell biological specialization that separates this subset from other DCs. Regardless of subset, DCs are collectively set apart from other cell types by an exquisite capacity for antigen acquisition and T cell stimulation. In this regard the tremendous rate of macropinocytosis by MDDCs coupled with very high expression of class II MHC may partly account for their ability to rescue some peptides for presentation to T cells despite the very proteolytic nature of their lysosomes [12]. Additionally, *in vivo* these cells are found predominantly at active inflammatory sites and may be particularly well suited for the acquisition, processing, and presentation of bulky particulate and microbial antigens more so than soluble proteinaceous antigens. This contrasts with other DC subsets which, while exhibiting a similar capacity for degradation of non-proteinaceous material, would easily preserve T cell epitopes from either a particulate or soluble source. Indeed, our previous studies using murine DCs demonstrate that they have a reduced capacity for the degradation of yeast as compared to murine macrophages [7].

As highly degradative cells, MΦs have a clear function in innate immunity, in wound healing, and in the effector arm of adaptive immunity where they participate in antigen clearance and in microbial killing and digestion. Native immunologically relevant antigens consist of biological macromolecules that must be degraded prior to presentation to T cells. *Prima facie* it is counterintuitive that the antigen presenting cells best equipped to stimulate T cells are poorly degradative, yet this underscores that *partial* degradation of antigens is an unequivocal requirement for the production of cognate T cell epitopes [23]. Indeed, though degradative cells have an ancient role in wound healing and innate immunity, the onset of adaptive immunity drove the need for a specialized cell type capable of preserving small peptides in the context of otherwise degradative lysosomes [28], [29].

## Materials and Methods

### Antibodies

The following antibodies were used for western blotting: mouse anti-human CatB (Serotec), rabbit anti-human CatD (Dako), goat anti-human CatL (Santa Cruz Biotechnology), rabbit anti-human CatS (CalBioChem), sheep anti-human AEP (R&D Systems), rabbit anti-human GILT (a kind gift of P. Cresswell, Yale University), and mouse anti-human γ-tubulin (Sigma). The anti-human monoclonal antibodies used for flow cytometry were as follows: anti-CD1a, -CD11c, -CD86, -CD123, -HLA-DR, and Lin1 (Lineage cocktail 1, a cocktail of antibodies directed against CD3, CD14, CD16, CD19, CD20, and CD56) (BDBiosciences).

### Cell Isolation and Culture

Human monocytes were isolated from buffy coats of healthy donors (New York Blood Bank) using RosetteSep Human Monocyte Enrichment Cocktail (StemCell Technologies) according to the manufacturer's protocol. For MDDC cultures, monocytes were grown at 1×10^6^cells/mL in 10 cm bacteriological-grade petri dishes (BD Biosciences) in RPMI 1640 supplemented with 10% FBS, 2 mM L-glutamine, 100 U/mL penicillin, 100 µg/mL streptomycin, 20 µg/mL gentamicin, (Gibco/Invitrogen), 150 ng/mL GM-CSF (Leukine (sargramostim), Bayer HealthCare Pharmaceuticals), and 25 ng/mL IL-4 (Peprotech) at 37°C. Immature MDDCs were harvested at day 5. For mature cells, maturation was induced on day 5 by adding 100 ng/mL lipopolysaccharide (LPS, Sigma) or DH5α bacteria (Stratagene) and allowing maturation to proceed for 24 hours.

For monocyte-derived MΦ cultures, monocytes were grown at 1×10^6^cells/mL in 10 cm bacteriological-grade petri dishes (BD Biosciences) in RPMI 1640 supplemented with 10% FBS, 2 mM L-glutamine, 100 U/mL penicillin, 100 µg/mL streptomycin, 20 µg/mL gentamicin (Gibco/Invitrogen), and 50 ng/mL M-CSF (Peprotech) at 37°C. MΦs were harvested on day 7.

CD34DCs were derived from CD34^+^ hematopoietic progenitor cells as previously described [13]. Briefly, purified CD34^+^ cells (generously provided by D. Krause, Yale University) were cultured at a density of 4×10^5^cells/mL in X-VIVO 10 medium (Cambrex) supplemented with 2 mM L-glutamine, 100 U/mL penicillin, 100 µg/mL streptomycin (Gibco/Invitrogen), 100 ng/mL GM-CSF (Leukine (sargramostim), Bayer HealthCare Pharmaceuticals), 20 ng/mL SCF, 2.5 ng/mL TNFα, 0.5 ng/mL TGFβ1, and 100 ng/mL Flt3L (Peprotech) at 37°C. After 7–10 days of culture the clustered cells were purified over a 7.5% BSA density cushion at 1xg for 30 minutes on ice. The pellets were retrieved and washed with cold PBS. The cells were then either taken as immature CD34DCs or were matured by culturing them in the growth medium supplemented with either 100 ng/mL LPS (Sigma) or DH5α bacteria (Stratagene) and allowing maturation to proceed for 48 hours.

Blood MDCs and PDCs were isolated as previously described [14]. Briefly, mononuclear cells were first isolated from buffy coats (New York Blood Bank) on a Ficoll-Paque gradient (GE Healthcare). The samples were enriched for DCs using a negative selection enrichment cocktail (EasySep Human Pan-DC Pre-enrichment Kit, StemCell Techologies) according to the manufacturer's protocol. The cells were labeled with Lin1, anti-CD123, anti-HLA-DR, and anti-CD11c and were sorted by FACS where the DCs were separated according to their phenotype. MDCs were Lin1^−^, HLA-DR^+^, CD11c^+^, CD123^−^. PDCs were Lin1^−^, HLA-DR^+^, CD11c^−^, CD123^+^.

### Preparation of Cell Lysates and Immunoblotting

As many lysosomal proteases are inactivated by alkaline or neutral conditions [30], [31], [32], the preparation of cell lysates was consistently performed in a slightly acidic buffer. The cell-free extracts were prepared in sucrose buffer (0.25 M sucrose, 20 mM HEPES, 2 mM EDTA (Sigma), pH 6.5) with 1% Triton X-100 (Sigma).

Gel electrophoresis and coomassie staining were performed according to standard protocols. Immunoblotting was performed with the indicated antibodies following SDS-PAGE and transfer to nitrocellulose filters (Schleicher and Schuell). All secondary antibodies used for western blotting were conjugated to HRP and the membranes were developed using an enzymatic chemiluminescence system (Pierce Biotechnology).

### Quantitative RT-PCR

Total RNA was isolated using the RNeasy Kit (Qiagen) according to the manufacturer's recommendations. Quantitative real-time RT-PCR was performed using the QuantiTect SYBR Green One-Step RT-PCR Kit (Qiagen) and detected with the Mx3000P® QPCR system (Stratagene). The data were normalized to the level of GAPDH expression in each individual sample. The ratio of transcript abundance was calculated using the immature CD34DC values as a base unit equal to one, thus allowing for display of the data as “fold-greater” than the immature CD34DCs.

### Lysosomal pH Measurements

Studies of lysosomal pH in intact cells were performed using an acidotrophic probe that selectively partitions into the lysosomal compartments of living cells. The probe used (LysoSensor Yellow/Blue DND-160, Molecular Probes) consists of a dye with two distinct optimal pH sensitivities, which allows dual-emission measurements and ratiometric quantitation of lysosomal pH. The procedure used for lysosomal pH measurements was adapted from the Molecular Probes Handbook [33], previous work by Haugland and colleagues [34], and previous work by Poole and colleagues [18]. This approach required a minimum of 18×10^6^ cells in suspension. 2×10^6^ cells were aliquoted out for use as a *blank* in later pH measurements. The staining medium containing 5 µM LysoSensor probe in 5 mL growth medium was allowed to equilibrate to 37°C in a water bath for 30 minutes. The remaining 16×10^6^ cells were pelleted and resuspended in the staining medium and placed at 37°C for 5 minutes. After incubation the cells were washed once with cold growth medium, twice with cold PBS, and resuspended in cold PBS. The cells were then divided into 8 separate aliquots and pelleted as was the *blank* sample separated above. All subsequent steps were done quickly and on ice.

A series of Mes/HEPES pH buffers were previously prepared by mixing Mes buffer (50 mM Mes, 50 mM NaCl, 30 mM Ammonium Acetate, 40 mM Sodium Azide (Sigma), pH 4.0) with HEPES buffer (50 mM HEPES, 50 mM NaCl, 30 mM Ammonium Acetate, 40 mM Sodium Azide (Sigma), pH 7.5) to achieve buffers of varying pH, ranging from pH 4.0 to pH 7.4.

Five of the LysoSensor-labeled aliquots were used for lysosomal pH calibration and were each resuspended in a Mes/HEPES variable pH buffer with one of the following levels of acidity: pH 4.0, pH 4.5, pH 5.0, pH 5.5, and pH 6.0. The remaining aliquots were resuspended in Mes/HEPES buffer, pH 7.4. This first resuspension in Mes/HEPES buffer was used as a wash and after centrifuging the aliquots, each was resuspended in 2 mL (1×10^6^ cells/mL) of the corresponding Mes/HEPES buffer.

Two minutes prior to fluorescence measurements of the samples, nigericin and monensin (CalBioChem) were added to a final concentration of 10 µM to the aliquots used for pH calibration. This allowed lysosomal pH to equilibrate with the Mes/HEPES buffer and facilitated the creation of a standard curve correlating lysosomal pH with the magnitude of fluorescence emission.

Fluorescence intensity of all samples was measured with a fluorescence spectrophotometer (Perkin Elmer) at an excitation wavelength of 360 nm and at two emission wavelengths: 451 nm and 518 nm. The *blank* sample was used for background subtraction at all wavelengths. Using the data from the pH calibration samples, a standard curve was calculated by plotting (em_451_/em_518_) vs. pH. This standard curve was used to back-calculate the lysosomal pH of the experimental samples from their emission values.

### *In vitro* Protein Degradation Assays

Protein degradation assays were developed in house to assess the proteolytic capacity of lysates from different cell types. Ovalbumin (OVA; CalBioChem) was used for degradation assays at a concentration of 0.5 mg/mL and cell lysates at a concentration of 1 mg/mL. Reactions were performed in degradation reaction buffer (0.1 M citrate, 1 mM EDTA, 2 mM DTT, 1% Triton X-100 (Sigma), pH 4.5) or control buffer (0.1 M Tris, 1 mM EDTA, 2 mM DTT, 1% Triton X-100 (Sigma), pH 7.4) at 37°C for 30 or 60 minutes. Samples were separated by SDS-PAGE and visualized by coomassie staining.

Real-time degradation assays were performed using self-quenching fluorophore-conjugated casein protein (BODIPY TR-X casein, Molecular Probes). Reactions proceeded in degradation reaction buffer (0.1 M citrate, 1 mM EDTA, 2 mM DTT, 1% Triton X-100 (Sigma), pH 4.5) at 37°C. The labeled casein was used at a concentration of 50 µg/mL and cell lysates were used at 0.25 mg/mL. Fluorescence intensity data was gathered at 10-second intervals using a plate reading fluorescence spectrophotometer (Molecular Devices) with an excitation wavelength of 589 nm and emission wavelength of 617 nm.

### Other Acid Hydrolase Activity Assays

The following hydrolase substrates were used at concentrations of 6 mM: 4-methylumbelliferyl-phosphate for acid phosphatase activity, 4-methylumbelliferyl-β-D-glucuronide for β-glucuronidase activity, and 4-methylumbellifery-α-D-mannopyranoside (Sigma) for α-mannosidase activity. Cell lysates were used at a concentration of 0.5 mg/mL in degradation reaction buffer (0.1 M citrate, 1 mM EDTA, 1% Triton X-100 (Sigma), pH 4.5) or control buffer (0.1 M Tris, 1 mM EDTA, 1% Triton X-100 (Sigma), pH 7.4). Reactions proceeded at 37°C for 60 minutes and were stopped with 0.4 M carbonate buffer (pH 9.0). Fluorescence intensity was measured on a fluorescence spectrophotometer (Perkin Elmer) with an excitation wavelength of 365 nm and emission wavelength of 450 nm.

### Endocytosis of HRP Immune Complexes and HRP Activity Assays

HRP immune complexes were formed by incubation of HRP or FITC-HRP (Roche Applied Science) with rabbit anti-HRP (Jackson ImmunoResearch Laboratories) at a mole:mole ratio of 1 mol HRP to 6.8 mol anti-HRP. DCs were pulsed with the 5 µg/mL HRP immune complexes for 2 hours, washed three times and chased for 30 minutes.

The immune complex-loaded cells were split into four different samples. The first sample was assessed for maturation markers by FACS and the second was used for an HRP activity assay as detailed below. The third and fourth samples were placed back into culture with either DC growth medium alone or DC growth medium plus a maturation stimulus (LPS or DH5α bacteria). After 24 hours (MDDCs) or 48 hours (CD34DCs) of incubation the samples were assessed for maturation markers by FACS and used for HRP-activity assay.

For measurement of HRP activity, DCs loaded with HRP-ICs were washed three times with PBS and dispensed (in 100 µL PBS) into microtiter plates (BDBiosciences). To each well 100 µL TMB substrate (3, 3′, 5, 5′-tetramethylbenzidine, Pierce Biotechnology) was added and OD(650) absorbance readings were acquired for each well at 10 second intervals using a plate-reading UV/Vis spectrophotometer (Molecular Devices).
